# Are Preschool-Aged Children Meeting Physical Activity Guidelines? A Systematic Review Covering 43,000 Participants Worldwide

**DOI:** 10.3390/healthcare14070869

**Published:** 2026-03-28

**Authors:** Markel Rico-González, Adrián Moreno-Villanueva, Iago Portela-Pino, Jorge Olivares-Arancibia, Ricardo Martín-Moya

**Affiliations:** 1 Department of Didactics of Musical, Plastic and Corporal Expression, University of the Basque Country, EHU, 48940 Leioa, Spain; 2 Department of Health Sciences, University Isabel I of Castille, 09003 Burgos, Spain; 3 Department of General and Specific Didactics and Theory of Education, University of León, 24007 Leon, Spain; iagoportt92@gmail.com; 4 AFySE Group, Research in PA and School Health, School of Physical Education, Faculty of Education, Universidad de las Américas, Santiago 72810, Chile; jorge.olivares.ar@gmail.com; 5 Department of Physical Education and Sports, Faculty of Education and Sport Sciences, Campus of Melilla, University of Granada, 52006 Melilla, Spain; ricardo.martinmoya@gmail.com

**Keywords:** education, health, World Health Organization, healthcare, children

## Abstract

**Background**: Since sedentary habits have become a growing global public health concern, the promotion of physical activity (PA) from early childhood could help children live healthy lifestyles. The aim of this systematic review was to analyze the level of compliance with PA in preschoolers in relation to the reference guidelines. **Method**: A systematic review of relevant articles was carried out using four databases (PubMed, ProQuest, SCOPUS, and FECYT (Web of Sciences, CCC, CIDW, KJD, MEDLINE, RSCI, and SCIELO)) until 14 May 2025. The methodological assessment process was performed by using an adapted version of the MINORS assessment criteria. **Results**: A total of 623 studies were initially found and 23 were included in the qualitative synthesis. **Conclusions**: The results revealed that the average in most contexts usually ranges between 30% and 65% of the child population. Due to different operational criteria, compliance was generally higher when PA was assessed separately using single-behavior guidelines as opposed to when integrated 24 h movement frameworks were used. However, these results should be considered with caution because establishing the level of adherence to PA guidelines is difficult due to the different outcomes and guidelines used to compare the level of children’s PA. In future research, it is important to establish common baseline criteria (specifying more specific ages, common questionnaires, and criteria for calculating PA quantity and intensity) to facilitate more objective and reliable comparisons between studies. This systematic review is important because it highlights the need for healthy educational habits from the first years of a person’s life.

## 1. Introduction

Physical activity (PA), which is a vital part of early childhood development, affects a number of physiological, cognitive, and socioemotional aspects of health [1]. Habits related to PA can set the stage for future health outcomes and behaviors during a child’s early years. In fact, it lowers the risk of non-communicable chronic diseases like type 2 diabetes, metabolic syndrome, and cardiovascular pathologies [2,3,4,5], encourages physiologically related factors (e.g., lower fat mass or higher bone mass index) [5,6,7,8], and supports mental health, socialization, and emotional intelligence [9,10,11].

Unfortunately, during the COVID-19 pandemic, the sedentary lifestyle was aggravated [12]. This situation increased the risk of children and adolescents developing respiratory and cardiovascular diseases [13] and also lower well-being and life satisfaction [14], among other variables influencing quality of life. In addition, it is possible that the long duration of periods of confinement due to the pandemic may have caused imbalances in the return to the daily routine of PA in children [14]. In this sense, it is likely that the daily PA of this population group has decreased as a consequence of the acquisition of more sedentary habits typical of the pandemic period [15].

For these reasons, and considering that years ago some authors declared that up to 81% of children were inactive globally [16], the rising frequency of sedentary lifestyles and associated health disorders in children has made PA particularly interesting [17]. In this way, to understand how much PA preschool-aged children perform, some institutions from different countries worldwide have proposed PA guidelines. Health recommendations for the promotion of PA in the kindergarten context could guide society in general and families and teachers in particular to create environments that promote active lifestyles from early childhood. These guidelines can be found in the document published by the World Health Organization (WHO) in 2020 [18], which are aligned in general terms with several PA recommendations in countries such as the United Kingdom [19], the United States [20], Australia [21], Canada [22] and Spain [23].

Preschool-aged children, often described as children up to 6 years old, represent a crucial demographic given the significance of PA because habits formed during this time can influence behavior patterns for the rest of one’s life [24]. In this sense, PA guidelines focused on these children of these ages divide recommendations depending on age within this age range: infants (<1 year), toddlers (1–2 years), and preschoolers (>3 years). In this regard, and aimed at establishing an overview, some guidelines, such as the WHO or Canadian 24 h movement guidelines, recommend >30 min in the tummy time position, >180 min of PA, and >180 min of PA (60 min should be moderate-to-vigorous PA (MVPA)), respectively [25].

Further objective measurement devices [25], questionnaires offer practical advantages for large-scale surveillance across diverse populations and settings [26], particularly in resource-limited contexts. To date, most previous reviews have focused on overall PA levels rather than specifically examining adherence to established guidelines, which is critical for public health surveillance and policy evaluation. Given the importance of early childhood as a foundational period for establishing lifelong PA habits, and considering the global variations in preschool PA patterns influenced by cultural, environmental, and socioeconomic factors [27,28,29,30], a comprehensive synthesis of questionnaire-based studies assessing guideline compliance in this age frame is important. Therefore, the aim of the present systematic review was to analyze the degree of compliance with PA habits in preschoolers recorded by questionnaires in relation to the recommended guidelines.

## 2. Materials and Methods

### 2.1. Followed Guidelines and Registration

This systematic review was conducted in accordance with the Preferred Reporting Items for Systematic Reviews and Meta-Analyses (PRISMA) guidelines [31] and followed the established protocols for conducting systematic reviews in the field of sport sciences [32]. The review protocol was crafted to guarantee thorough examination of the pertinent literature while upholding methodological precision. This systematic review was registered in PROSPERO: CRD420261306520.

### 2.2. Information Sources

A thorough search was performed across four primary databases: PubMed, SCOPUS, Fundación Española para la Ciencia y la Tecnología (including Web of Sciences, Current Contents Connect (CCC), Current Index to Web of Science (CIDW), Korean Journal Database (KJD), MEDLINE, Russian Science Citation Index (RSCI), and SCIELO), and ProQuest Central. The search included all the available literature published before 14 May 2025. This selection of databases was made to guarantee extensive coverage of the literature in both medical and sports science fields.

### 2.3. Search Strategy

The PICO (Patient, Problem, or Population–Intervention or Exposure–Comparison, Control, or Comparator–Outcome[s]) framework and the PECOS structure (Population, Exposure, Comparison, Outcomes, and Study Design) were utilized to organize the search approach and ensure thorough coverage of the pertinent literature. To ensure clarity, the authors were aware of the journal names and the authors of the manuscripts. Filters for language were applied to include only publications in English and Spanish. The search terms were meticulously chosen to encompass all the relevant literature on adherence to reference guidelines through questionnaires in preschool environments. The final search query was:

(Preschool OR kindergarten OR “early childhood”) AND (“meet*”) AND (guidelines) AND (“physical activity”)

### 2.4. Eligibility Criteria

The researchers implemented a search string in various databases and collected the titles, author names, journal information, and publication dates of all articles retrieved. After structuring the Excel spreadsheet, any duplicates were eliminated, and the articles that remained were assessed for their eligibility according to inclusion/exclusion criteria (Table 1). In cases where the authors discovered articles that were not included in the initial search, these were added to the Excel document as “included from external sources.”

### 2.5. Data Extraction

An Excel spreadsheet created in compliance with the Cochrane Consumers and Communication Review Group’s data extraction template was used to conduct a consistent data extraction procedure [33]. The spreadsheet made it easier to evaluate the inclusion and exclusion criteria for each of the chosen research works in a methodical manner. The extraction procedure was carried out independently by two authors (M.R.-G. and A.M.-V.), with any disputes being settled by discussion until an agreement was reached. For publications that were excluded, complete documentation was kept, along with detailed justifications. Every piece of information was methodically documented and kept in the spreadsheet. After checking the information in all the included studies, all the data were extracted and are classified depending on the reference guideline that was used, and detail the information of participants (country, age, and institutions for recruitment).

### 2.6. Assessment of Study Methodology

The methodological index for non-randomized studies (MINORS) was used to evaluate the methodological quality [34]. When the topics being examined are comparative, the MINORS scale, which consists of eight essential points, is extended to twelve points. In this instance, nine items (out of 18 points) were evaluated because three of them could not be applied (NA). These three items were considered as “NA” because they are used to evaluate controlled trials with at least two groups. These terms are: adequate control group (item 9), simultaneous groups (item 10), and homogeneous starting groups (item 11). Depending on the quality of each point, each part may receive a score ranging from 0 to 2 (Table 2).

## 3. Results

### 3.1. Identification and Selection of Studies

**Figure 1:**
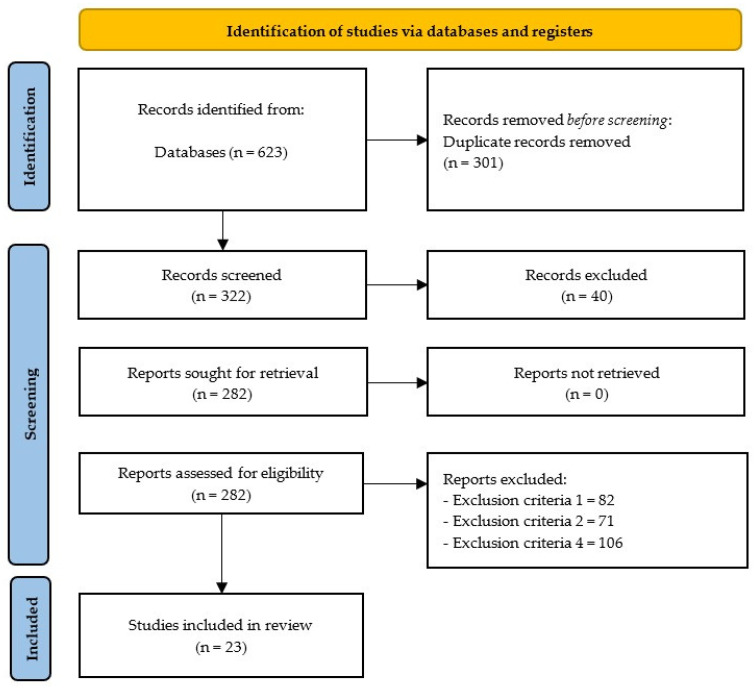
PRISMA flow diagram.

After analyzing all databases (Web of Science: 103; PubMed: 123; ProQuest: 88; SCOPUS: 309), 301 duplicate articles were first found when the contents of 623 articles were examined. Exclusion criterion number one (*n* = 82), exclusion criterion number two (*n* = 71), exclusion criterion number four (*n* = 106), and exclusion criterion number five (*n* = 40) were applied to eliminate 245 articles after the authors examined whether each of the remaining 322 articles satisfied all inclusion criteria. The qualitative summary of the systematic review comprised the remaining 23 articles (Figure 1).

### 3.2. Methodological Quality

Table 2 contains the quality assessment for this systematic review.

Although the MINORS tool was primarily used to describe the methodological quality of the included studies, its results were also considered when interpreting the findings of the review. Overall, most studies showed moderate to high methodological quality, with MINORS scores ranging from 16 to 18 points (Table 2), which supports a reasonable level of confidence in the reported compliance rates. However, the recurrent methodological limitations identified—particularly those related to the use of self-reported questionnaires [35,36,37,38,39,40,41,42,43,44,45,46,47,48,49], the absence of objective validation measures, and the lack of prospective sample size estimation [50]—were taken into account when interpreting the heterogeneity of the results. Consequently, studies were not differentially weighted based on their MINORS scores, but higher-quality studies were considered to provide more robust estimates, while findings from other studies with comparatively lower scores were interpreted with greater caution [51]. This approach allowed the quality assessment to inform the narrative synthesis by contextualizing the strength and reliability of the evidence, rather than functioning solely as a procedural checklist.

**Table 2:** Methodological assessment of the included studies.

Reference	1	2	3	4	5	6	7	8	9	10	11	12	Score
McGowan et al. [35]	2	2	2	2	2	2	2	NA	NA	NA	2	2	18/18
Hesketh et al. [36]	2	2	2	2	2	2	2	NA	NA	NA	1	2	17/18
Gonçalves et al. [37]	2	2	2	2	2	2	1	NA	NA	NA	2	2	17/18
Ramírez-Vélez et al. [52]	2	2	2	2	2	2	2	NA	NA	NA	2	2	18/18
Chia et al. [38]	2	2	2	2	2	2	2	NA	NA	NA	2	2	18/18
Chia et al. [39]	2	2	2	2	2	2	1	NA	NA	NA	2	2	17/18
Prioreschi Micklesfield, [40]	2	2	2	2	2	2	1	NA	NA	NA	2	2	17/18
Pujadas Botey et al. [41]	2	2	2	2	2	2	2	NA	NA	NA	1	2	17/18
Fukushima et al. [42]	2	2	2	2	2	2	2	NA	NA	NA	2	2	18/18
Gao et al. [43]	2	2	2	2	2	2	2	NA	NA	NA	2	2	18/18
Xiong et al. [53]	2	2	2	2	2	2	2	NA	NA	NA	1	2	17/18
Nejadsadeghi et al. [50]	2	2	2	2	2	2	2	NA	NA	NA	1	2	17/18
Jekauc et al. [44]	2	2	2	2	2	2	2	NA	NA	NA	1	2	17/18
Byrd-Williams et al. [45]	2	2	2	2	2	2	2	NA	NA	NA	2	1	17/18
Vanderloo et al. [46]	2	2	2	2	2	2	2	NA	NA	NA	2	2	18/18
Okely et al. [51]	2	2	2	2	2	1	2	NA	NA	NA	2	1	16/18
Loprinzi et al. [54]	2	2	2	2	2	2	1	NA	NA	NA	2	2	17/18
Lin et al. [55]	2	2	2	2	2	2	2	NA	NA	NA	2	2	18/18
Quah et al. [56]	2	2	2	2	2	2	2	NA	NA	NA	2	2	18/18
Joan et al. [57]	2	2	2	2	2	2	2	NA	NA	NA	2	2	18/18
Loh et al. [47]	2	2	2	2	2	2	2	NA	NA	NA	2	2	18/18
Rivera et al. [48]	2	2	2	2	2	2	2	NA	NA	NA	2	2	18/18
Amornsriwatanakul et al. [49]	2	2	2	2	2	2	2	NA	NA	NA	2	2	18/18

**Note:** NA = not applicable.

### 3.3. Study Characteristics

The 23 studies included in this systematic review show that the results on the degree of compliance with the recommendations are mixed. In several studies, between 20% and 40% of participants met the minimum PA recommendations [35,36,37,41,45,46,47,48], while other studies reported compliance rates of more than 50% [37,38,40,42,43,51,54]. On the other hand, studies were observed where less than 20% of the children complied with the recommendations [44,46,49,50,55,56,57]. This variability can be attributed to, among other factors, the country of origin of the study, the age group considered (within the 0–6-year interval), the sex of the participants, the time of day or week when PA was measured, and to whom the questionnaire was addressed (parents vs. children). In this sense, studies that applied questionnaires to parents tended to report higher compliance rates, suggesting a possible overestimation [38,41,42,43,47,48,51,54].

However, these results should be considered with caution because establishing the level of adherence to PA guidelines is difficult due to two issues: instruments and reference guidelines.

First, different validated questionnaires were used across studies, which may partly explain variability in reported compliance rates. The most commonly used questionnaires were adapted versions of the Youth Risk Behavior Survey [35], SMALLQ^®^ [38,39], and questionnaires from entities such as the WHO [35,38,39,44,48,49,52,57], the Canadian Society of Exercise Physiology (CSEP) [40,41,43,46,53], and the National Association of Physical Education of the USA (NASPE) [45,51,54,55]. This methodological diversity limits the possibility of establishing direct comparisons between studies.

Second, the included studies were grouped according to the specific normative framework used to evaluate compliance, as summarized in Table 3. A substantial proportion of the studies adopted an integrated 24 h movement guideline approach, in which PA, sedentary behavior (screen time), and sleep were jointly assessed within a single behavioral framework, primarily based on the WHO 24 h movement guidelines and their national adaptations [35,36,37,38,39,40,43,46,47,48,49,52,53,56]. This integrated approach was also reflected in country-specific implementations of the 24 h framework, including those applied in Australia [36,37], Canada [40,43,46,53], and Singapore [47,56]. In contrast, a second group of studies evaluated compliance exclusively against PA-only guidelines, focusing on daily minutes of total PA or moderate-to-vigorous PA, without integrating sleep or sedentary behavior into a combined criterion [44]. These PA-only frameworks included recommendations issued by the WHO (PA-specific guidelines) [44], as well as national and professional guidelines developed by organizations such as the National Association for Sport and Physical Education (NASPE) [45,51,54,55] and the Canadian Society for Exercise Physiology (CSEP) [40,43,46,53]. This distinction between integrated 24 h movement guidelines and PA-only guidelines was consistently applied in the organization of Table 3, allowing a clearer interpretation of the heterogeneity observed across studies and avoiding conceptual overlap between fundamentally different guideline frameworks.

The characteristics of studies were extracted and clustered into Table 3.

**Table 3:** The level of compliance with PA guidelines in preschoolers.

Ref.	Sample	Reference Guideline	Results/Compliance
**NASPE**			
Loprinzi et al. [54]	164 parents of preschoolers	The National Association of Sport and Physical Education (NASPE) Active Start Guidelines	Weekdays: 50% PA compliance Weekend: 65% PA compliance
Lin et al. [55]	264 children between 3 and 6 years	National Association for Sport and Physical Education Guidelines	10.2% met the NASPE PA guideline Participants spent an average of 155 min/week in low-intensity PA
Okely et al. [51]	266 preschool children’s parents in two large cities on the East Coast of Australia	National Association for Sport and Physical Education (NASPE) Guidelines	Weekdays: 56% PA compliance Weekend: 79% PA compliance
Byrd-Williams et al. [45]	475 preschool (2–5 years) centers	National Association for Sport and Physical Education (NASPE) Guidelines	20.72% met the NASPE recommendation (>90–120 min daily)
**OTHER AMERICAN GUIDELINES**			
Nejadsadeghi et al. [50]	120 preschool children (4–6 years) in southwest Iran	American Medical Association Guidelines	Only 2.5% met the guideline of 60 min of structured PA every day
**WHO 24 h GUIDELINES**			
Rivera et al. [48]	1145 parents of children (<5 years)	WHO 24 h Guidelines on PA, Sedentary Behaviour, and Sleep	81.6% met the PA guideline 20.1% met all 24 h guidelines
Amornsriwatanakul et al. [49]	1051 parents of children (3–6 years)	WHO 24 h Guidelines: Global Recommendations on PA for Health	48.0% met the PA guideline 14.6% met all variables (PA, sleep and ST)
Ramírez-Vélez et al. [52]	3002 low-income preschoolers (3–4 years)	24 h World Health Organization Guidelines	21.4% met the PA guideline
Chia et al. [39]	1481 parents/legal guardians of children (2–5 years)	2019 WHO Integrated 24 h Guidelines	31.0% met all variables (PA, sleep and ST)
Chia et al. [38]	2066 parents of preschoolers	24 h World Health Organization Guidelines	56.9% met the PA guideline
McGowan et al. [35]	123 children (2–5 years) in mid-Michigan	24 h Movement Behavior Recommendations Set by the World Health Organization (2019)	20.3% met the PA recommendation
Joan et al. [58]	939 preschoolers (3–6 years)	WHO 24 h Movement Guidelines	67.1% met the PA recommendation
**WHO GUIDELINES (no 24 h, PA only)**			
Jekauc et al. [44]	989 preschoolers (4–5 years)	WHO (PA only): Global Recommendations on PA for Health	15.3% met the PA recommendation (13.1% of girls and 17.4% of boys)
**SINGAPORE INTEGRATED 24 h GUIDELINES**			
Loh et al. [47]	267 parents of children (0–2 years)	Singapore Integrated 24-h Activity Guidelines for Early Childhood	37.3% of infants and 20.6% of toddlers met PA guidelines
Quah et al. [56]	303 preschoolers	Singapore Integrated 24-h Activity Guidelines for Early Childhood	Weekend: 78.9% PA compliance Weekdays: 55.4% PA compliance
**AUSTRALIAN 24 h MOVEMENT GUIDELINES**			
Hesketh et al. [36]	455 infants, with a mean age of 3.6 months	Australian 24 h Movement Guidelines	29.7% met PA guidelines
Gonçalves et al. [37]	324 families from a rural district in northeastern Brazil	Australian 24 h Movement Guidelines	47.5% met the PA recommendation
**CANADIAN 24 h MOVEMENT GUIDELINES**			
Xiong et al. [53]	Preschoolers (2–4 years)	Canadian 24-h Movement Guidelines	Meeting screen time and PA guidelines was associated with greater quality of life improvements; the associated improvements with guideline adherence were lowest in the 2–4-year old group
Gao et al. [43]	10,967 preschoolers (3–6 years)	Canadian 24-h Movement Guidelines	62.3% met the moderate-to-vigorous PA (MVPA) recommendation
Prioreschi Micklesfield, [40]	119 preschoolers from Soweto, South Africa	Canadian 24 h Movement Guidelines for the Early Years	58% met PA guidelines
Vanderloo et al. [46]	767 preschoolers (3–4 years)	The Canadian 24-h Movement Guidelines	23.1% met PA guidelines
**OTHER CANADIAN GUIDELINES (not 24 h, PA only)**			
Pujadas Botey et al. [41]	631 parents and their children	Canadian PA and Sedentary Behaviour Guidelines (not 24 h, PA only)	38% met PA guidelines
**JAPANESE GUIDELINE (not 24 h, PA only)**			
Fukushima et al. [42]	821 children (3–6 years) from Unnan city, Shimane prefecture	Japanese PA Guideline	65.7% met the PA recommendation (70.2% of boys and 61.2% of girls)

**Note:** PA (PA), ECE (early care and education centers), NAP SACC (Nutrition and Physical Activity Self-Assessment for Child Care), moderate-to-vigorous physical activity (MVPA), physical activity (PA), screen time behavior (ST), sleep behavior (SLP), World Health Organization (WHO), and National Association for Sport and Physical Education (NASPE).

## 4. Discussion

Although PA guidelines for children have a similar conceptual structure, the research analyzed in our review shows that there are significant differences in terms of operational criteria, prioritized components, and methods for assessing compliance.

### 4.1. Differences and Similarities Among the Various PA Guidelines Referenced

Regarding the differences and similarities among the various PA guidelines referenced, first, a clear similarity among the included studies is the adoption of the 24 h integrated approach, which combines PA, sedentary behavior (especially screen time), and sleep [36,37,38,43,47,48,52,56,57,58]. Convergence towards this model suggests a progressive homogenization of international benchmarks. In all these contexts, compliance is usually assessed individually by component and in an integrated manner [37,43,48,49,52,56]. However, differences emerge in the operationalization of PA. Some studies focus their analysis on adherence to total daily volume (≥180 cumulative minutes), while others emphasize time spent in moderate-to-vigorous PA (MVPA) as the main criterion [35,37,40,42].

Another important distinction lies in the methodology of measurements. Some studies utilize objective accelerometry [35,37,40,42], whereas others rely on self-reported or parent-reported questionnaires [38,43,57]. This difference significantly impacts direct comparisons between countries. According to the empirical evidence provided by the studies analyzed, objective measurements yield better results than parent-reported questionnaires, especially with regard to screen time and PA intensity [35,37,40].

Regarding sedentary behavior, screen time is the main indicator of sedentary behavior in most of the guidelines reviewed [39,43]. Nevertheless, specific limits can vary based on the version of the guidelines adopted or the country. Furthermore, similarities are observable in the factors associated with compliance. Several analyses concur that parenting practices and the family environment have a significant impact on adherence to the guidelines [37,39,45]. These similarities indicate that, despite differences in national regulations, the social factors of compliance exhibit similar patterns across nations.

Finally, it is worth noting that some studies highlight the connection between compliance and findings in cardiometabolic health or cognitive development [35,41,46,53], while others focus more on political or environmental variables [45].

Overall, the reviewed articles show a clear international convergence toward an integrated 24 h movement model, but they also reveal methodological, cultural, and contextual differences in the implementation and evaluation of the recommendations.

### 4.2. The Percentage of Compliance Associated with Each Guideline

The current evidence shows a significant variation in adherence rates to PA recommendations for preschool-aged children across studies and contexts.

First, for the WHO’s proposed PA guidelines, moderate compliance is typically observed, ranging from 40% to 65%, especially when the total volume of daily activity is used as the main criterion [18,41,43]. In contrast, adherence to the WHO’s 24 h movement guidelines, when focusing on the PA component within this integrated framework, is generally somewhat lower, ranging from 45% to 60%, reflecting the influence of more rigorous operational definitions and broader behavioral requirements [35,37,38,46,47,48,49,52,56,57]. Similar moderate levels of adherence are demonstrated by national adaptations of the 24 h framework for early childhood. It estimates variations by age group and measurement method, ranging from roughly 30–40% in infants and toddlers to roughly 50–60% in preschool-aged children [36,47,48,56,57].

Regarding the degree of compliance of preschoolers with PA, it is moderate and varies depending on the context. Hesketh et al. [36] reported that 30–40% of children meet PA guidelines in Australia, while Gonçalves et al. [37] detected a prevalence near 47% among preschool children in rural areas in Brazil. Ramírez-Vélez et al. [52] reported that approximately 56% of Colombian children met the specific PA component. Gao et al. [43] detected a prevalence of approximately 62% in China, and analyses carried out in Singapore indicated similar figures (between 50% and 60% compliance) [47,56]. In Southeast Asia, Amornsriwatanakul et al. [49] estimated that around 56% of preschool children in Thailand meet the recommendations. According to studies conducted in Japan [42] and South Africa [52], compliance is at more moderate levels, between approximately 40% and 50%. However, data from Canada indicate slightly higher figures, around 65%, when PA is analyzed without considering other behaviors [41,46]. These results indicate that, despite significant diversity between nations, about 1/3 to 2/3 of preschool children manage to follow the recommendations for PA.

These results also imply that methodological factors (e.g., the type of measurement tool used, the definition of intensity thresholds, and whether activity is measured as total accumulated movement or specifically as moderate-to-vigorous PA) have a significant impact on reported compliance.

### 4.3. Comparisons Across Countries or Guideline Frameworks

Regarding the comparisons across countries or guideline frameworks, it should be clarified that PA suggests that intensity can be assessed in relative and absolute terms [20] and that these may vary depending on the country, historical moment, or even political context in which it was performed [59].

The “24 h movement guidelines” framework, which combines sleep, PA, and sedentary behavior, is used in places such as Australia [36,48], Singapore [38,47,56], Brazil [37], Colombia [52], China [43], and Thailand [49]. However, compliance rates and distribution by component vary between countries. For example, in Australia [36,48], the PA component generally shows fairly high percentages, while screen time limits overall compliance. Similar behavior is detected in Singapore [47,56], where adherence to the PA component is higher than to sedentary behavior. In Latin America, it has been observed that in both Brazil [37] and Colombia [52], combined reference guidelines and analyses show a lower PA level in children. Although the framework in China is aligned with international recommendations [43], compliance rates vary depending on regional and sociodemographic factors, showing internal differences within the country itself. This occurs in all nations and may be the result of disparities that can be observed within the same country [60,61].

Research conducted in Singapore [38,47,56] and Malaysia [47] indicates that, although there is similarity in the adoption of integrated guidelines in Southeast Asia, there are differences in levels of compliance linked to cultural and family factors. The study of behaviors outside the school context in Thailand [49] shows additional variations that are not always reflected in research conducted in Western contexts.

Furthermore, comparisons show discrepancies in methodological frameworks. While other studies use parent questionnaires (from China [43], Malaysia [57] and Singapore [38,56]), some studies employ objective accelerometry to determine compliance (from Japan [42], South Africa [40] and Brazil [37]). These discrepancies in terms of methodology have an impact on comparability between countries and may have repercussions for compliance estimates. Furthermore, research examining associations with health or development outcomes [35,41,46,53] indicates that the way in which the effect of guidelines is understood may vary depending on the country and the study design. On the other hand, studies focusing on institutional policies and environmental factors [45,62] point out that the structure of the environment has an impact on the level of effective implementation of guidelines. This helps to understand disparities between countries beyond formal normative content.

Overall, adherence to PA guidelines differs depending on the framework used for the guidelines as well as geographical location. Moderate adherence is usually seen when PA is evaluated separately, usually falling between roughly 40% and 65%. For instance, when total daily activity was taken into account, studies in China and Canada found relatively higher compliance levels within this range [41,43], while more moderate estimates (ranging from 40% to 50%) have been noted in South Africa and Japan under country-specific PA guidelines [40,42]. In contrast, studies applying the WHO 24 h movement guidelines in regions such as Brazil, Colombia, Singapore, and Thailand report somewhat lower adherence to the PA component, generally between 45% and 60%, reflecting the broader behavioral requirements of this integrated framework [37,38,47,48,49,52,56,57]. The national adaptations of the 24 h guidelines show similar trends in Australia and Southeast Asia, where compliance with PA guidelines varies by age group (infants and toddlers: 30–40%; preschoolers: 50–60%) [36,47,48,56,57]. These findings show that a variety of factors, such as the guidelines’ conceptual framework, local lifestyles, and environmental factors, affect adherence levels.

However, further to these results, there is disagreement when comparing among countries the level of compliance, even within the same country [60,61]. Therefore, further large-scale, intercontinental studies are needed to determine whether preschool-aged children meet PA recommendations [63].

## 5. Limitations

A notable limitation of this study is the reliance on questionnaire-based assessments of PA, completed by parents, caregivers, and children, resulting in the need to interpret these results with caution. Although the authors acknowledge the potential overestimation of PA reported by parents, they do not critically address the reliability of data provided by preschool children, which raises concerns regarding the validity of the findings. In addition, some other limitations should be considered, such as reliance on subjective questionnaires, recall and social desirability bias, lack of comparability across guideline frameworks, and absence of objective PA validation.

Additionally, preschool PA recommendations can be divided into three main groups: (a) a total of 180 min per day of active behavior (at any intensity; total PA- TPA) is recommended; (b) of these 180 min, a total of 60 min of moderate–vigorous PA (MVPA) per day is recommended; (c) the recommendation takes into account the context of the PA, i.e., whether it is performed in the context of structured or unstructured PA. And, according to the WHO, the recommendations for young children (0–5 years) are more differentiated and differ from those written by the authors. Following WHO guidelines, infants (under 1 year of age), children aged 1 to 2 years, children aged 3 to 4 years, and older children are considered separately. A limitation in this study is that we cannot highlight the compliance with different guidelines because the articles do not differentiate these groups, maybe because the authors only report the level of compliance, although they take into account these differences. However, we encourage the reader to interpret the conclusions of this systematic review with caution because defining the age group of children between 0 and 6 years as a sample could lead to misinterpretation of the data from the outset.

Finally, although we selected the most-used words in the search strategy, it should be analyzed if adding words such as attain*, adherence, or compliance (in addition to meet*) or recommendation* (in addition to guidelines) could provide more articles for inclusion.

## 6. Conclusions

In conclusion, while the conceptual underpinnings of PA guidelines for preschool-aged children are generally similar, especially the broad use of integrated 24 h movement frameworks, significant variations exist in their operational criteria, methods of measurement, and application in various contexts. Applying integrated guidelines led the authors to find a significant decrease in compliance with PA recommendations, which is generally moderate when viewed in isolation, typically falling between 30% and 65%. Due to the latter’s more comprehensive behavioral requirements, compliance with WHO PA recommendations is generally higher than that with WHO 24 h movement guidelines. The main obstacle to complete adherence is still the sedentary component, particularly screen time, while PA by itself seems to be more manageable. Moreover, cross-national comparisons show that no country consistently performs better than others in every component because methodological variations, sociocultural elements, environmental circumstances, and national modifications to the guidelines all affect compliance levels. In addition, differences in measurement approaches, intensity thresholds, and age-group classifications contribute to variability in reported adherence across studies. Since assessment instruments and age-group definitions vary amongst studies, the results should be interpreted with caution. Overall, the data point to the need for context-specific approaches and more standardized international monitoring to increase the comparability and efficacy of future guidelines, even though a sizable percentage of preschoolers attain the recommended PA levels.

## Figures and Tables

**Table 1:** Inclusion and exclusion criteria.

N	Item (PICOS/PECOS)	Inclusion	Exclusion	Search String
1	Population	Healthy children from preschools, kindergartens, or early childhood (0–6 years) Studies with national and international samples were included	Children moving on from preschool (such as to elementary school, middle school, high school, bachelor’s programs, or undergraduate studies) or children who were not healthy	Preschool OR kindergarten
2	Intervention/Exposure	Measuring PA using questionnaires, but during usual circumstances	Not measuring PA using questionnaires or measuring during unusual circumstances (e.g., COVID-19 pandemic)	“PA”
3	Comparation	Comparing PA with guidelines	Articles that measured PA but did not compare it with the guidelines	Meet *
4	Outcome [s]	Measures related to PA Studies that report the level of compliance with the guidelines	Variables not related to PA Studies without results, such as protocols Studies that do not report the level of compliance with the guidelines	Guidelines
5	Other criteria	Full-text, peer-reviewed original research that was published in English or Spanish in a journal	Non-peer-reviewed journal articles Non-original full-text studies (conference papers…) Studies written in another language	

**Note** = PA: physical activity. * means significant difference.

## Data Availability

No new data were created or analyzed in this study. Data sharing is not applicable to this article.
